# Bacterial contamination upon the opening of injection needles

**DOI:** 10.1186/s40981-018-0197-7

**Published:** 2018-08-29

**Authors:** Shintaro Abe, Isao Haraga, Fumiaki Kiyomi, Hitomi Kumano, Akira Gohara, Shigehiro Matsumoto, Ken Yamaura

**Affiliations:** 1 Department of Anesthesiology, Fukuoka University Faculty of Medicine, 7-45-1 Nanakuma, Jonan-ku, Fukuoka-shi, Fukuoka, 814-0180 Japan; 2 Department of Anesthesia, Fukuoka University Chikushi Hospital, 1-1-1 Zokumyouin, Chikushino-shi, Fukuoka, 818-8502 Japan; 3 Statitician, Ph.D., Academia, Industry and Government Collaborative Research Institute of Translational Medicine for Life Innovation, Fukuoka University, 7-45-1, Nanakuma, Jonan-ku, Fukuoka-shi, Fukuoka, 814-0180 Japan

**Keywords:** Infection control, Catheter infection, Needle, *Staphylococcus aureus*

## Abstract

**Introduction:**

Two opening methods are used for injection needle products: the “peel-apart method” where the adhesive surface of the packaging mount is peeled off, and the “push-off top method,” where the needle hub is pressed against the mount to break it. However, the risks of bacterial contamination as a result of opening method remain unknown. The aim of our study was to evaluate the bacterial contamination of needle hubs upon the opening of injection needles by the peel-apart or push-off top method under various conditions.

**Methods:**

Bacterial contamination upon the opening of injection needles was examined in two materials, paper and plastic. Various concentrations of *Staphylococcus aureus* were applied to the mount and were maintained under wet or dry conditions. Injection needles were opened using the peel-apart or push-off top method. Needle hub contamination was examined using agar medium colony counting. Clinically assumed conditions (the hands and saliva of anesthesiologists) were also evaluated. Data were statistically examined using the Cochran-Mantel-Haenszel, Jonckheere, and Fisher’s exact tests.

**Results:**

The lateral surfaces of needle hubs were contaminated using the push-off top method, but not by the peel-apart method, in a manner that was dependent on *S. aureus* concentrations. No significant differences were observed between mount materials. Needle hub contamination was significantly more severe for the wet than for the dry opening portion. The clinically assumed condition study revealed that the lateral and bottom surfaces of the needle hub were contaminated significantly more in the saliva contamination group than in the dry and wet hand groups.

**Conclusions:**

The bacterial contamination of needle hubs may occur upon the opening of injection needles when the push-off top method is used and may be affected by hands contaminated with saliva under clinical conditions.

## Background

Bacterial contamination in infusion lines causes sepsis, resulting in prolonged artificial respiration and an extended stay in intensive care units or hospitals [1, 2]. The valves of infusion lines or syringes for drug injection are involved in bacterial contamination of infusion lines [3, 4]. Injection needles, which are used to aspirate drug solutions, may contaminate infusion lines through syringes when the syringes are contaminated with bacteria [5]. The present study focused on the contamination of needle hubs.

## Methods

### Opening methods

**Fig. 1:**
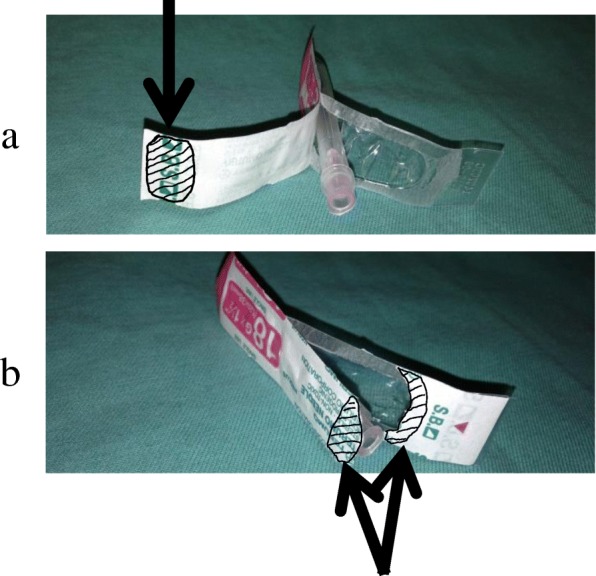
Opening methods. **a** Peel-apart method: opening by peeling the adhered planes of the blister and mount. **b** Push-off top method: opening by pressing the needle hub to the mount and breaking it. The arrow indicates the part at which the bacterial suspension was applied

Two injection needle product opening methods were employed: the “peel-apart method” (Fig. 1a) where the adhesive surface of the mount for packaging is peeled off, and the “push-off top method” (Fig. 1b) where the needle hub is pressed against the mount to break it [5].

### Bacterial strains and preparation of bacterial solutions

The methicillin-susceptible *Staphylococcus aureus* strain of the American Type Culture Collection (ATCC) 29213 was used [6]. ATCC 29213 was provided by the Kitasato University-Laboratory of Infection Control and Research Center. A bacterial solution was cultured for 10 h with shaking and was diluted with physiological saline to an absorbance of 0.3 using an absorption spectrometer at 578 nm [7]. The concentration of the bacterial suspension was 10^8^ colony-forming units/ml (CFUs/ml) before the experiment. This solution was diluted with physiological saline to six different concentrations (10^8^, 10^7^, 10^6^, 10^5^, 10^4^, and 10^3^ CFUs/ml).

### Experimental contamination of mounts

A total of 240 injection needles, including 120 each adherently packaged with a paper-mount and transparent plastic blister (18G: Terumo Co., Ltd., Tokyo, Japan) or with a plastic (combination of polystyrene and polyethylene terephthalate) mount and transparent plastic blister (18G: NIPRO Co., Ltd., Osaka, Japan) were used. The injection needle was taken out of the box just before the initiation of experiments and was stored on a clean bench after disinfection. Injection needles were classified into two groups according to the opening methods: the peel-apart and push-off top methods (60 needles each). Experiments were conducted separately for 10 needles each at six different concentrations of the bacterial suspension. To assess the risk of needle contamination by various quantitative concentrations under clinical settings, 10 μl of each of the bacterial suspensions (10^8^, 10^7^, 10^6^, 10^5^, 10^4^, and 10^3^ CFUs/ml) was applied to the part near the needle hub’s opening at the mount of an unopened injection needle product using a pipette tip on a clean bench (shaded parts in Fig. 1a, b). Using the peel-apart method, the bacterial suspension was applied to the gripped part of the mount (shaded parts in Fig. 1a). Using the push-off method, the bacterial suspensions were applied to the part potentially touching the mount when removing the needle (shaded parts in Fig. 1b).

Injection needles were then opened using the peel-apart or push-off top method with disinfected gloves (Fig. 1a, b). On a clean bench, half of the needles were opened as soon as the bacterial suspensions had been applied (wetness group). The other half were dried using the filtering airflow of the clean bench at room temperature. One hour later, the dry state of suspensions applied was confirmed visually and needles were then opened on the clean bench (dryness group). Injection needles were taken out to examine the degree of contamination in each part of the needle hub.

**Fig. 2:**
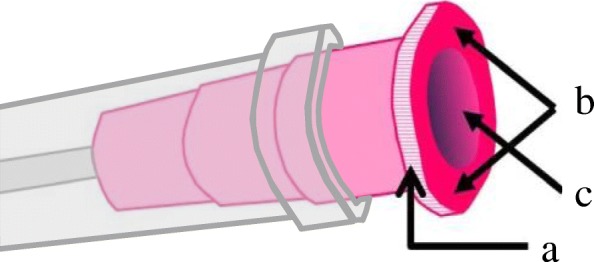
Contamination was evaluated in each region of the needle hub. **a** Lateral surface. **b** Bottom surface. **c** Inner lumen

To examine the degree of contamination on each site of the needle hub (Fig. 2), all lateral surfaces (Fig. 2a) were placed on agar medium and rotated to be brought into contact with the medium. The bottom surface of the needle hub (Fig. 2b) was then pressed against the agar medium. To examine contamination in the inner lumen (Fig. 2c), a 1-ml syringe containing 0.1 ml of saline was connected to the needle to discharge all saline onto the agar medium.

The agar medium was incubated at 37 °C for 30 h for colony counting. Brain heart infusion agar (Becton, Dickinson, and Company, USA) was used as the agar medium.

### Emulated clinical contamination (hand and saliva contamination of mounts)

Based on the rare occurrence of needle hub contamination in the previous experiment using the peel-apart method, various conditions (i.e., dry or wet hands, saliva contamination) were examined to evaluate the clinical risk of needle hub contamination using the push-off top method. Five anesthesiologists were included in the present study. This investigation was conducted in accordance with the current Declaration of Helsinki. The authors’ own samples were collected, and patients and volunteers were not included. All samples were anonymized after collection for the impossibility to identify the specific individual. A total of 150 injection needles, including 75 each in the paper-mount group and plastic-mount group, were used.

Anesthesiologists rubbed dry/wet hands on the paper or plastic-mounts without gloves. To simulate wet hands, 10 μl of autoclaved physiological saline was applied to dry hands using a micropipette. To simulate a hand contaminated with a patient’s saliva, gloved fingers licked by anesthesiologists were applied to each paper and plastic-mount.

Five saliva samples were obtained from each of the five anesthesiologists and were quantitatively cultured.

All injection needle products with clinically contaminated mounts were opened on a clean bench in the same manner as described for examination of the experimental contamination of mounts.

### Statistical analysis

The numbers of bacteria on the lateral surfaces, bottom surfaces, and total surface (sum of the lateral surface, bottom surface, and inner lumen) of needle hubs were compared between the opening methods, dryness/wetness of bacterial solution, and mount materials using the Cochran-Mantel-Haenszel test considering the concentration of *S. aureus* as a stratum. By comparing the dryness/wetness of bacterial solution and mount materials, only data obtained using the push-off top method was used because only one needle hub was contaminated in the peel-apart method. The trend test for the concentration of *S. aureus*-contamination relationship was performed using the push-off top method data with the Jonckheere test.

The number of bacteria in the inner lumen of a needle hub was classified into contaminated (≥ 1 colony) or uncontaminated (no colony), and this binary response was compared between opening methods, the dryness/wetness of the bacterial solution, and mount materials using Fisher’s exact test without considering the concentration of *S. aureus*. A trend test for the *S. aureus* concentration-contamination relationship in the number of bacteria in the inner lumen was not performed because of only five hubs were contaminated. Fisher’s exact test was instead applied to compare *S. aureus* concentrations.

Regarding emulated clinical contamination data, the number of bacteria was compared between mounts using the Cochran-Mantel Haenszel test considering the anesthesiologist and wet/saliva hands as strata, excluding dry hand data because all were zero. The numbers of bacteria on the lateral surfaces, bottom surfaces, inner lumens, and total surface of needle hubs were compared between dry/wet/saliva hands using the Cochran-Mantel-Haenszel test considering anesthesiologist as a stratum. The mount was not included into stratum because a large *p* value was obtained for the mount comparison. Pairwise comparisons were also performed. The family-wise error rate was controlled using the closed testing procedure; first, data was compared between dry, wet, and saliva hands with a significance level of 5% and the testing procedure was stopped if not significant. Second, pairwise comparisons were performed with a significance level of 5% for each test if the first step was significant. A *p* value of < 0.05 was considered to be significant. Data were analyzed using SAS version 9.4 (SAS Institute, Cay, North Carolina, USA).

## Results

### Opening methods

**Fig. 3:**
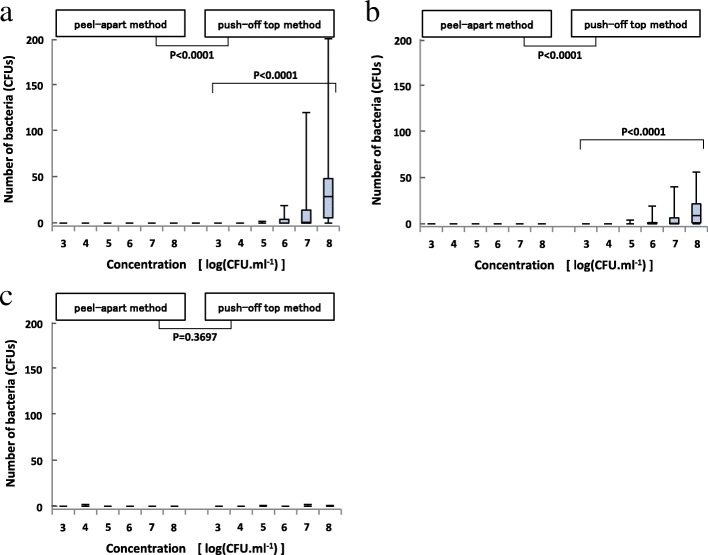
**a** Contamination of the lateral surface of the needle hub. **b** Contamination of the bottom surface of the needle hub. **c** Contamination of the inner lumen of the needle hub. Regarding (**a**) and (**b**), the Cochran-Mantel-Haenszel test considering the concentration of *S. aureus* as a stratum was used to compare between opening methods. The Jonckheere test was performed to evaluate the concentration of the *S. aureus*-contamination relationship for the push-off top method data and was not performed for the peel-apart method because only one needle hub was contaminated. Regarding (**c**), since fewer contaminated needle hubs were observed, the number of bacteria was classified into contaminated/uncontaminated, and Fisher’s exact test was used to compare opening methods

The lateral and bottom surfaces of needle hubs were contaminated significantly more by the push-off top method than by the peel-apart method (Figs. 3a, b). However, contamination of the inner lumen did not significantly increase (Fig. 3c).

### *S. aureus* concentrations

Using the push-off top method, contamination of the needle hub increased with the concentration of *S. aureus* applied to the opening portions. Contamination of the needle hub was rare at a concentration of ≤ 10^4^ CFUs/ml. The number of contaminated needle hubs was 5% (1 out of 20 needles) at a concentration of 10^4^ CFUs/ml, and 0% (0 out of 20 needles) at a concentration of 10^3^ CFUs/ml.

### Wet/dry

**Fig. 4:**
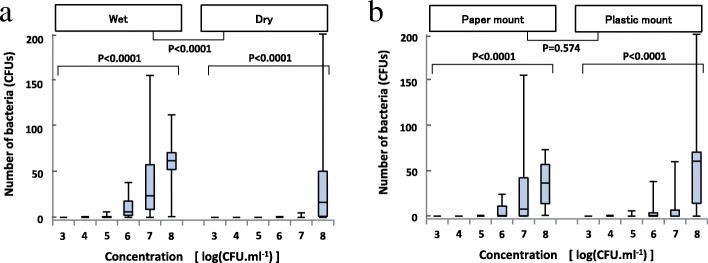
**a** Contamination of the needle hub in the push-off top method (wet/dry). **b** Contamination of the needle hub in the push-off top method (paper/plastic)

Contamination of the needle hub was significantly greater in the wet than in the dry opening portions (Fig. 4a).

### Paper/plastic

No significant differences were noted in the needle hub contamination between mount materials (paper and plastic) (Fig. 4b).

### Emulated clinical contaminations (hand and saliva contamination of mounts)

**Fig. 5:**
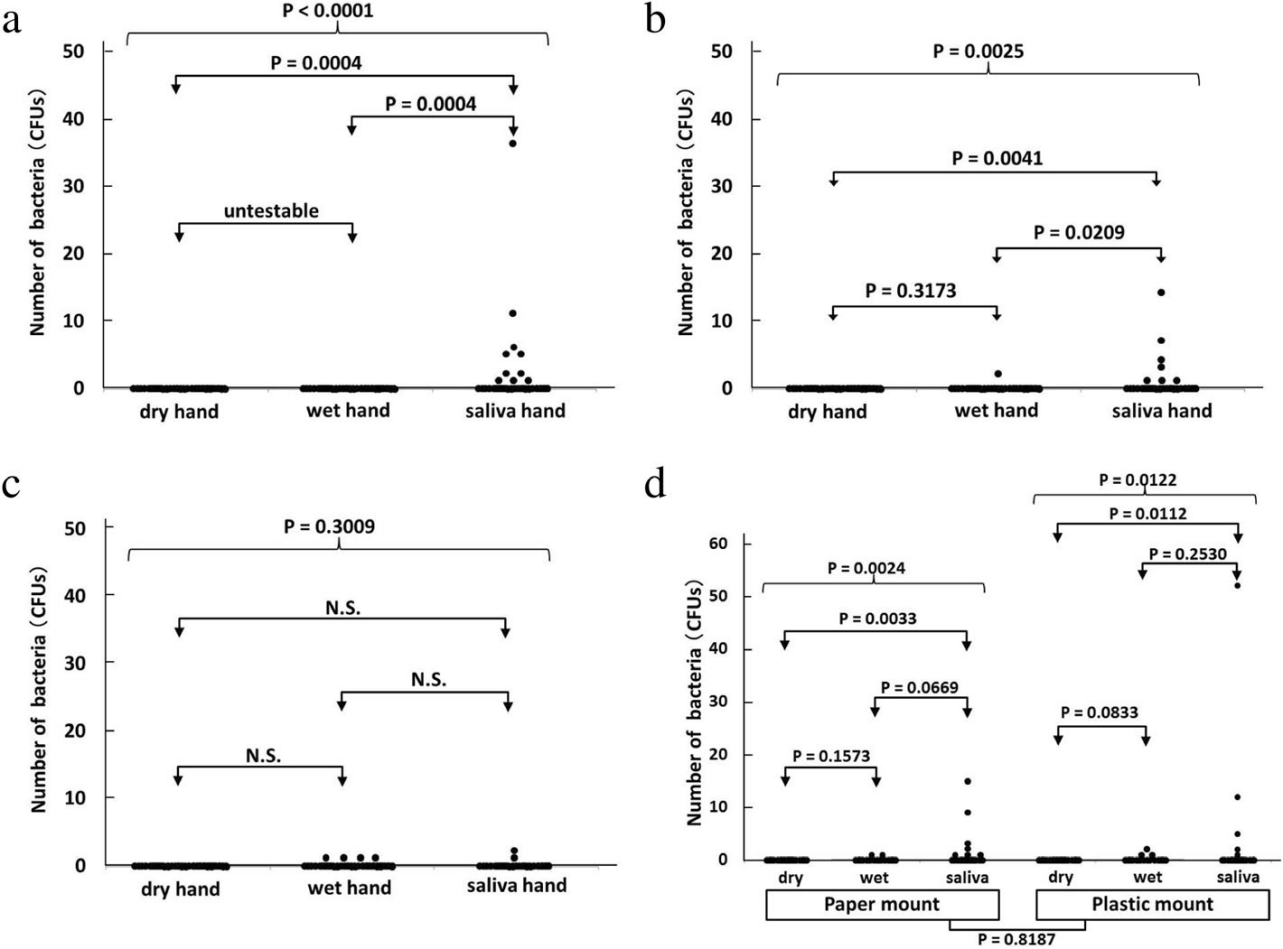
**a** Contamination of the lateral surface of the needle hub in the push-off top method (dry/wet/saliva). **b** Contamination of the bottom surface of the needle hub in the push-off top method (dry/wet/saliva). **c** Contamination of the inner lumen of the needle hub in the push-off top method (dry/wet/saliva). **d** Contamination of the needle hub in the push-off top method (paper/plastic). All bacterial data obtained under dry and wet conditions were zero, and, thus, a statistical test was not performed. The closed testing procedure was used. First, data was compared between dry, wet, and saliva hands with a significance level of 5%, and if not significant, we concluded that there are no significant differences for any pairwise comparisons and stopped the testing procedure. Second, pairwise comparisons were performed with a significance level of 5% for each test if the first step was significant. Three group comparisons were *p* = 0.0001, 0.0025, 0.3009, 0.0024, and 0.0122 for the lateral surface (**a**), bottom surface (**b**), inner lumen (**c**), paper-mount (**d**), and plastic-mount (**d**), respectively

No significant differences were observed in the needle hub contamination between the dry and wet hand contamination groups (Fig. 5a–c). The lateral and bottom surfaces of the needle hub were contaminated significantly more in the saliva contamination group than in the dry and wet hand groups (Fig. 5a, b). However, contamination of the inner lumen did not significantly increase (Fig. 5c). No significant differences were observed in the emulated clinical contamination of the needle hub between mount materials (paper and plastic) (Fig. 5d). The mean bacterial concentration of the saliva of five anesthesiologists was 2.36 × 10^7^ CFUs/ml (ranging between 2.2 × 10^6^ and 6.1 × 10^7^ CFUs/ml).

## Discussion

The present results showed that the risk of bacterial contamination was higher with the push-off top method than with the peel-apart method. Needle products are opened without contact between bacterially contaminated mounts and sterile needle hubs using the peel-apart method. However, needle products are opened due to rupture by pressing the needle hub to the mount using the push-off top method. The contaminated lateral and bottom surfaces of needle hubs appeared to be attributed to contact between bacterially contaminated mounts and sterile needle hubs.

We examined the type of bacteria that causes bacterial contamination as a related factor. We used *S. aureus* in the present study because it is one of the most frequently isolated pathogens from the epidermis and central line-associated bloodstream infections [8, 9]. Therefore, ATCC 29213 was selected as the standard strain because it exhibits an intermediate biofilm formation ability as an adhesion factor [10].

Gram-negative bacillus, particularly *Pseudomonas aeruginosa*, is a known cause of catheter-related bloodstream infections [9]. As with *S. aureus*, gram-negative bacilli may also cause contamination of a needle while a package is being opened. However, we did not include gram-negative bacilli in this study because the assays used to assess the biofilm formation and response pattern to drying and wetting in *S. aureus* cannot be performed easily in bacilli. Future studies are needed in this area.

We also examined the effects of various concentrations of *S. aureus* as one of the risk factors. Contamination of the lateral and bottom surfaces of the needle hub was enhanced by increasing concentrations of *S. aureus*. However, the inner lumen contamination did not significantly increase under wet conditions, even at 10^8^ CFUs/ml, which is the presumed concentration after exposure to human saliva [11].

As another related risk factor, we assessed the dryness or wetness of the bacterial solution.

Touch contact with wet hands led to an average of 6 × 10^4^ microorganisms translocating, whereas dry touch contact resulted in an average of 8.5 × 10^2^ microorganisms translocating [12].

Planktonic *S. aureus* under wet conditions may easily move with the flow of a liquid.

The minor flow of a liquid with an opening may result in the motion of liquid from the surface of the mount to the needle hub, causing bacterial movement.

Based on these findings, clinical conditions were examined. The causes of needle contamination were assumed to be the anesthesiologist’s hands with or without saliva. Since anesthesiologists have many opportunities to touch the intraoral saliva of a patient, human saliva is considered to be a colonizing source of puncture sites/needles [13]. Contaminated mounts due to a gloved finger with saliva contaminated needle hubs significantly more than in the hand contamination group.

The push-off method increased bacterial concentration, and wet needle mounts and hands may all contribute to needle hub bacterial infection.

As a limitation of the present study, the extrapolation of our results to clinical settings must be made with caution because our model was artificial. In the present study, the investigator who opened the needle mounts was the same investigator who applied the bacterial suspension. Explanations regarding inter-anesthesiologist variations have not yet been confirmed, but inter-anesthesiologist differences need to be considered. Therefore, contamination may occur due to the peel-apart method. However, the effects of inter-anesthesiologist variations appeared to be small because the opening procedure is a simple operation. Further studies with different bacteria and the repeated connection/disconnection of needles and syringes are warranted.

## Conclusions

These results indicate that the bacterial contamination risk of the push-off top method may occur upon opening of injection needles and may be affected by hands contaminated with saliva under clinical conditions.

## Abbreviations

ATCC: The American Type Culture Collection
CFUs: Colony-forming units
G: Gauge
